# Supplementary figures and images for: Patterns and functional consequences of antibody speciation in maternal-fetal transfer of coronavirus-specific humoral immunity
Source: PLoS Pathog. 2025 Aug 6;21(8):e1013408. doi: 10.1371/journal.ppat.1013408 (PMC12349702; doi:10.1371/journal.ppat.1013408)

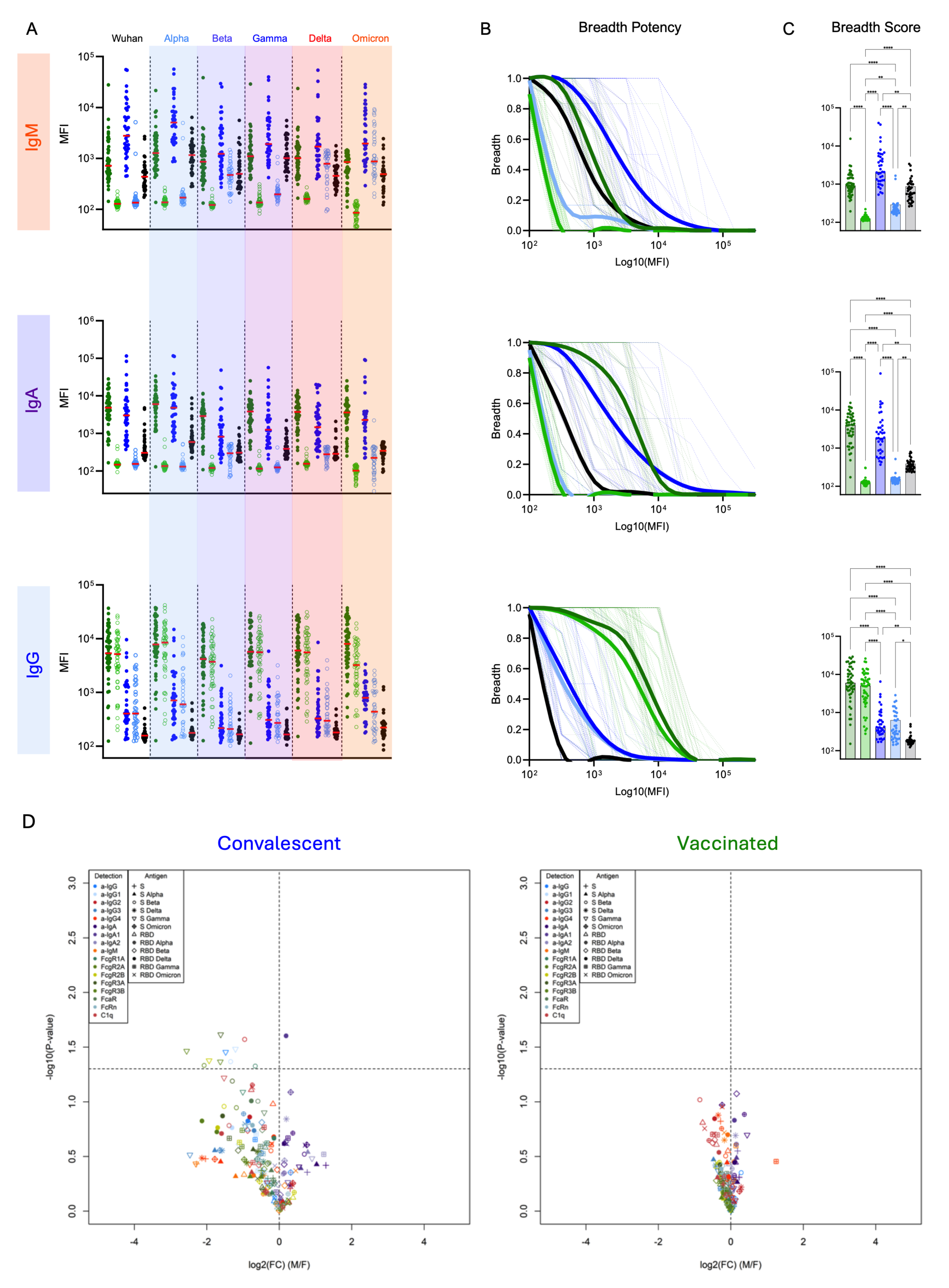

Supplement: S1 Fig — A. IgM, IgA, and IgM antibody binding responses in maternal (filled) and cord (open) samples among convalescent (n = 38) (blue) or vaccinated (n = 50) (green) individuals against SARS-CoV-2 RBD antigens. Naïve subjects (n = 37) are shown in black. B. Breadth-potency curves represent the fraction of subjects with a response exceeding a given level for IgM, IgA, and IgG antibody responses across the panel of VOC. Population means are shown with a thick line, and individual subjects illustrated in thin lines. C. IgM, IgA, and IgG breadth scores for each subject. Bar indicates the median. Statistical significance was defined by ANOVA Kruskal–Wallis test with Dunn’s correction and α = 0.05 (*p < 0.05, **p < 0.01, ***p < 0.001, ****p < 0.0001). D. Volcano plots presenting the fold-change (x-axis) and statistical significance (Mann Whitney test, y-axis) of differences in cord blood antibody profiles between male and female neonates born to convalescent (left) and vaccinated (right) mothers. Antibody isotype is indicated by shape, with RBD and whole spike indicated in hollow and filled symbols, respectively. SARS-CoV-2 variant is indicated by color. (TIFF) [file ppat.1013408.s001.tiff]

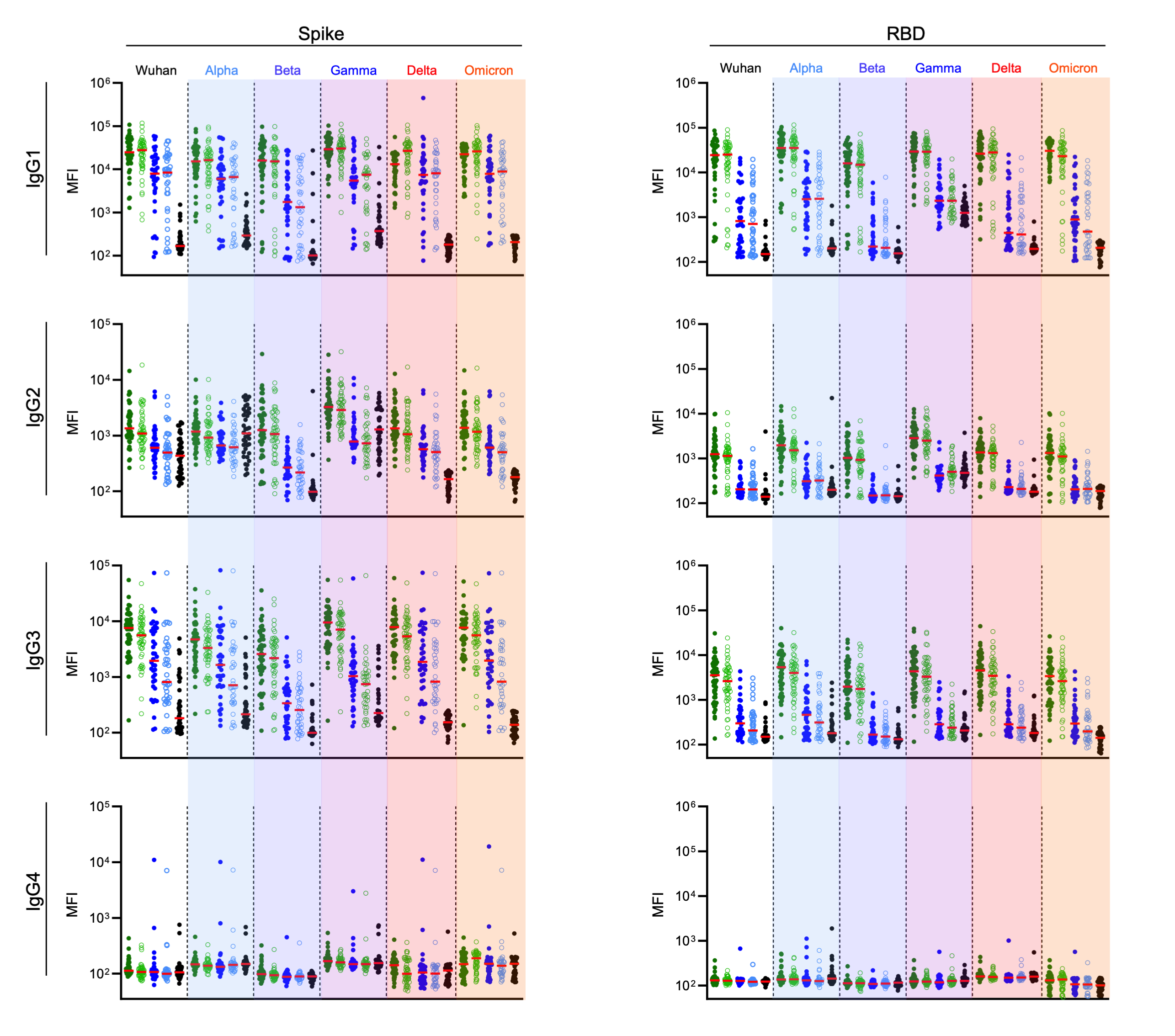

Supplement: S2 Fig — IgG subclasses binding activities toward spike and RBD-specific antibodies across Wuhan and VOC proteins in serum for maternal (filled) and cord (open) samples among convalescent (n = 38) (blue) or vaccinated (n = 50) (green) individuals. Naïve (n = 37) (black) subjects. Bar indicates median. (TIFF) [file ppat.1013408.s002.tiff]

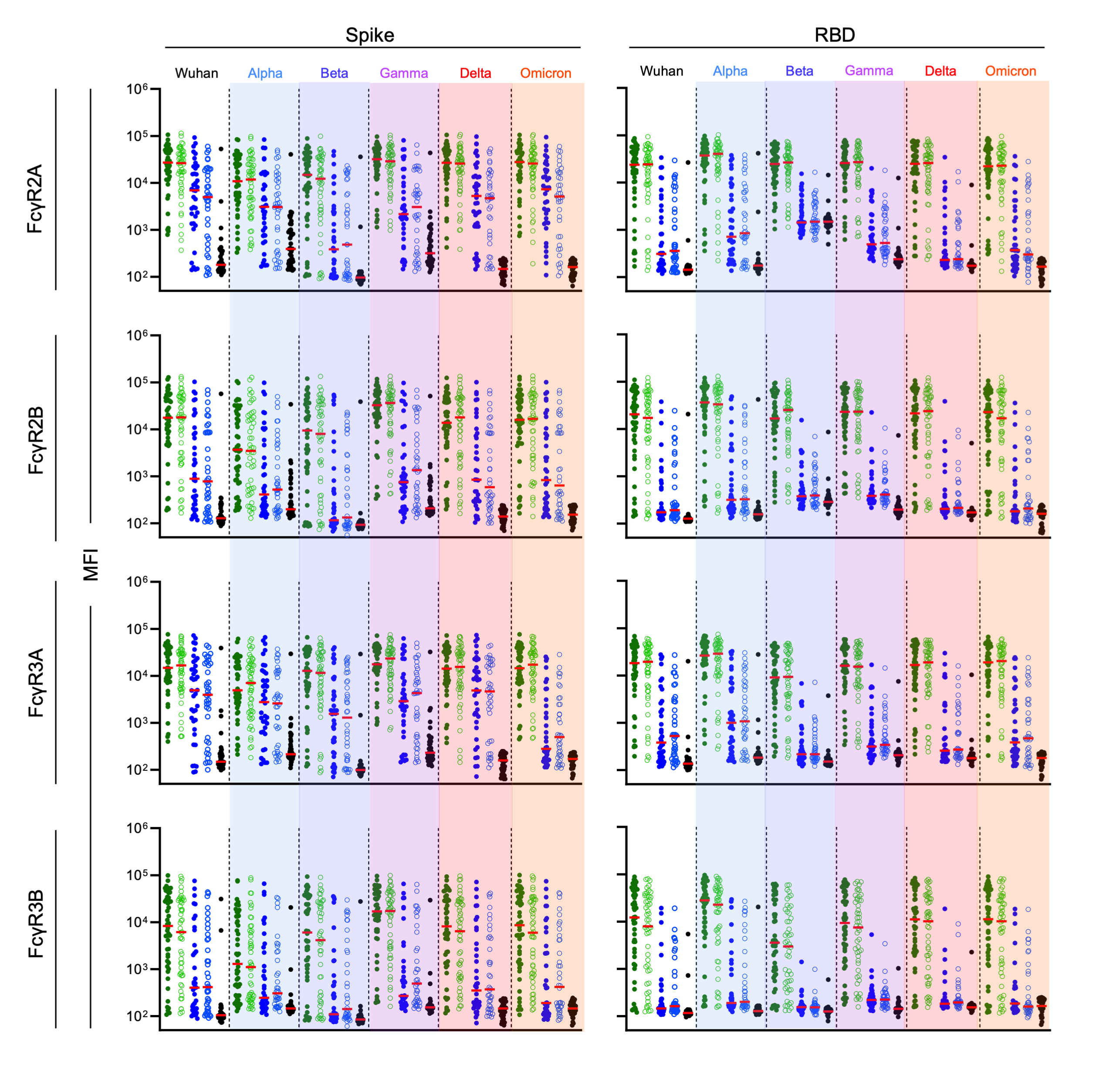

Supplement: S3 Fig — Fc receptor binding activities of spike- (left) and RBD- (right) specific antibodies across Wuhan and VOC proteins in serum in maternal (filled) and cord (open) samples among convalescent (n = 38) (blue) or vaccinated (n = 50) (green) individuals. Naïve (n = 37) (black) subjects. Bar indicates median. (TIFF) [file ppat.1013408.s003.tiff]

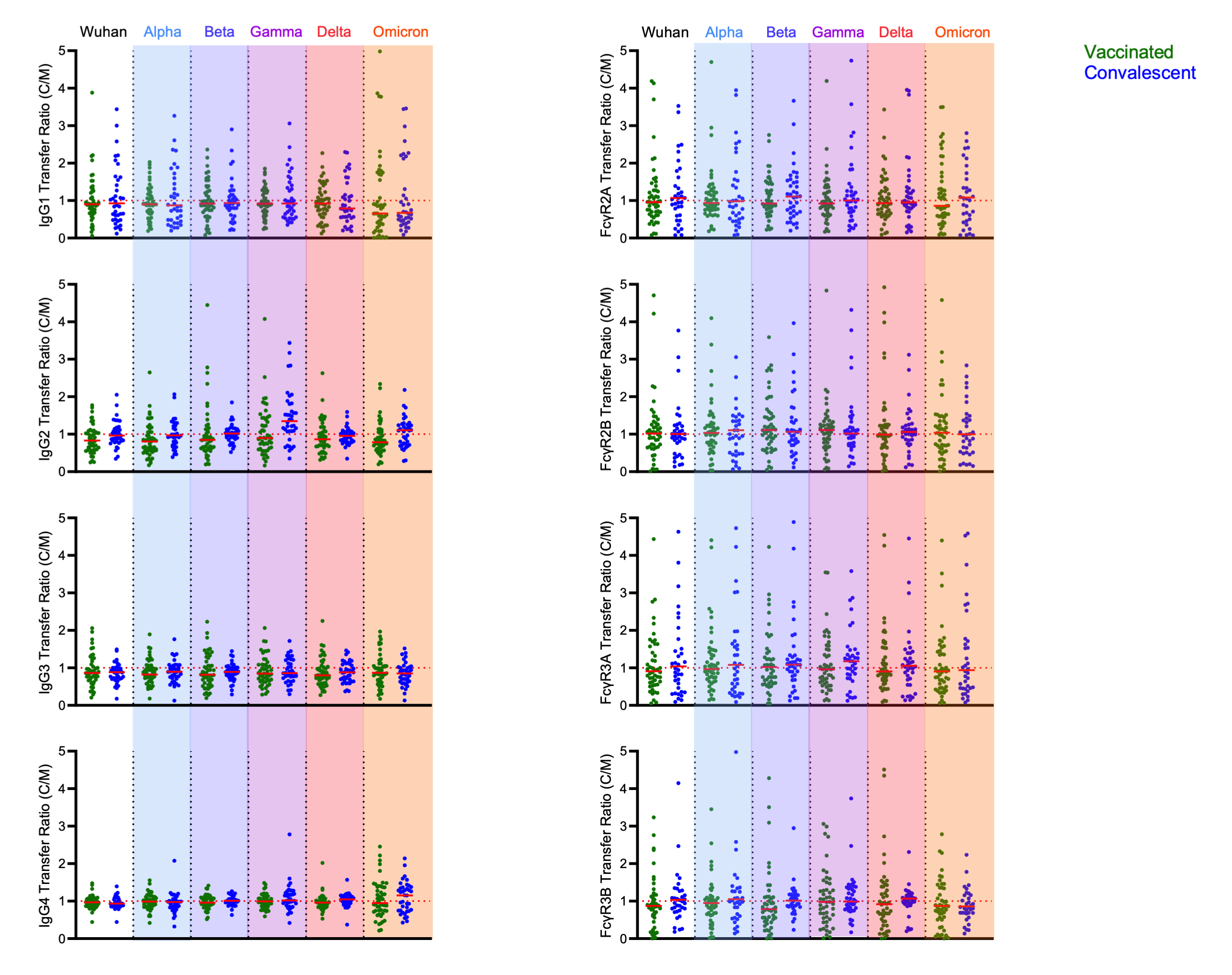

Supplement: S4 Fig — Transfer ratio (cord/maternal levels) SARS-CoV-2 RBD-specific IgG subclasses (left) and Fc receptor antibody binding (right) in vaccinated (green) and convalescent (blue) dyads. Bar indicates median. (TIFF) [file ppat.1013408.s004.tiff]

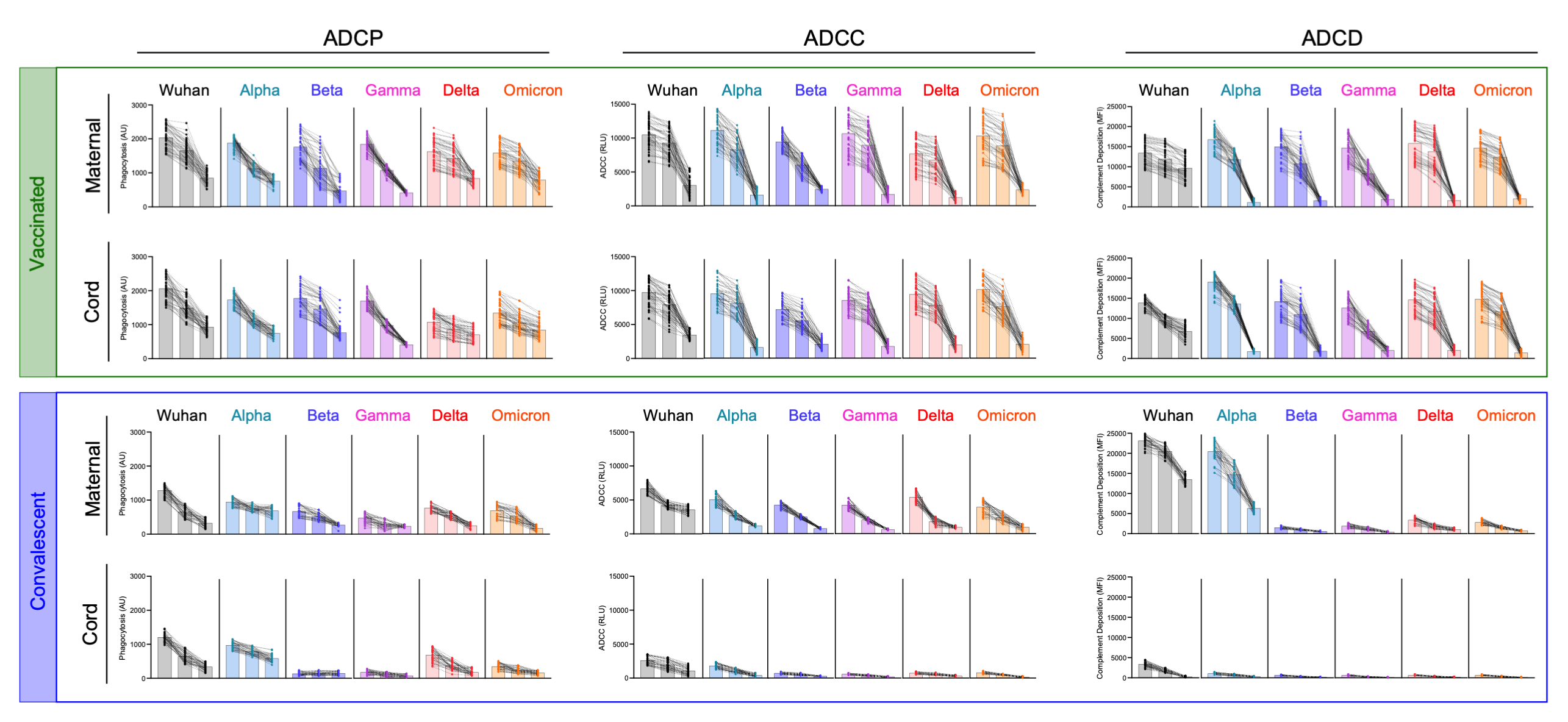

Supplement: S5 Fig — Ab effector functions from vaccinated maternal and cord blood (top, green box) and convalescent maternal and cord blood (bottom, blue box) for SARS-CoV-2 spike variants. Phagocytosis (left), ADCC (center), and Complement Deposition (ADCD, right) activities were assessed at each of three serum dilutions (1:50, 1:100, 1:250). Individual traces for each subject across dilutions are displayed. Functional activity is reported in arbitrary units (AU), relative light units (RLU), and median fluorescent intensity (MFI). Bar indicates median. (TIFF) [file ppat.1013408.s005.tiff]

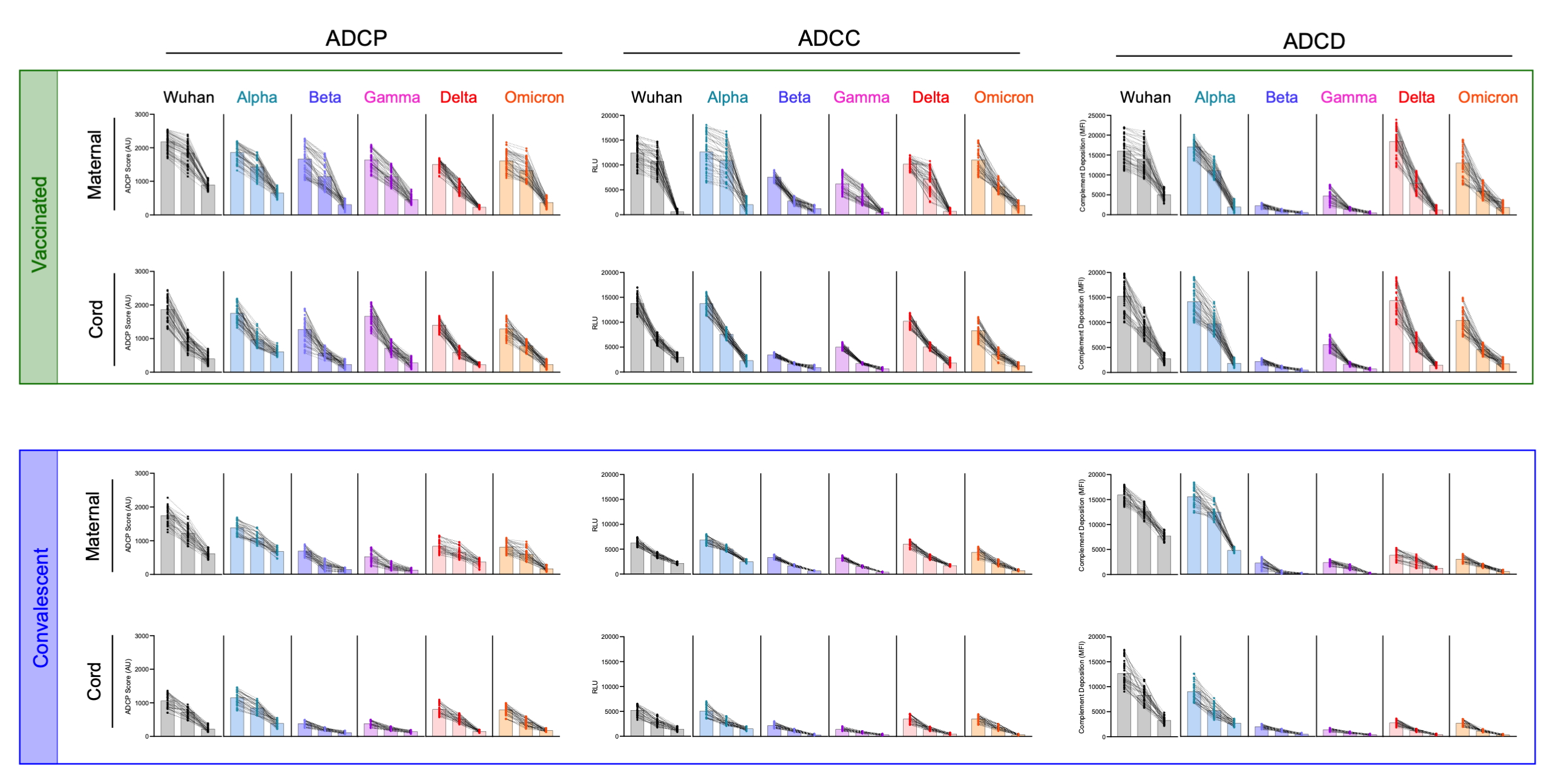

Supplement: S6 Fig — Ab effector functions from vaccinated maternal and cord blood (top, green box) and convalescent maternal and cord blood (bottom, blue box) for SARS-CoV-2 RBD variants. Phagocytosis (ADCP, left), ADCC (center), and Complement deposition (ADCD, left) activities were assessed at each of three serum dilutions (1:50, 1:100, 1:250). Individual traces for each subject across dilutions are displayed. Functional activity is reported in arbitrary units (AU), relative light units (RLU), and median fluorescent intensity (MFI), respectively. Bar indicates median. (TIFF) [file ppat.1013408.s006.tiff]

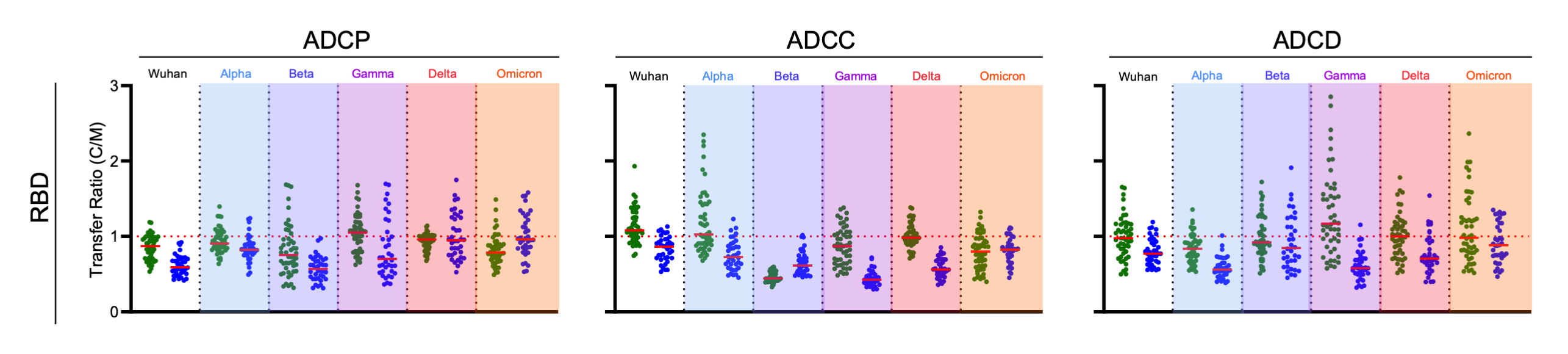

Supplement: S7 Fig — Transfer ratio (cord/maternal levels) of antigen-specific (SARS-CoV-2 VOC RBD antigens) Fc effector functions including ADCP (left), ADCC (center) and ADCD (right) in vaccinated (green) and convalescent (blue) dyads. Bar indicates median. (TIFF) [file ppat.1013408.s007.tiff]

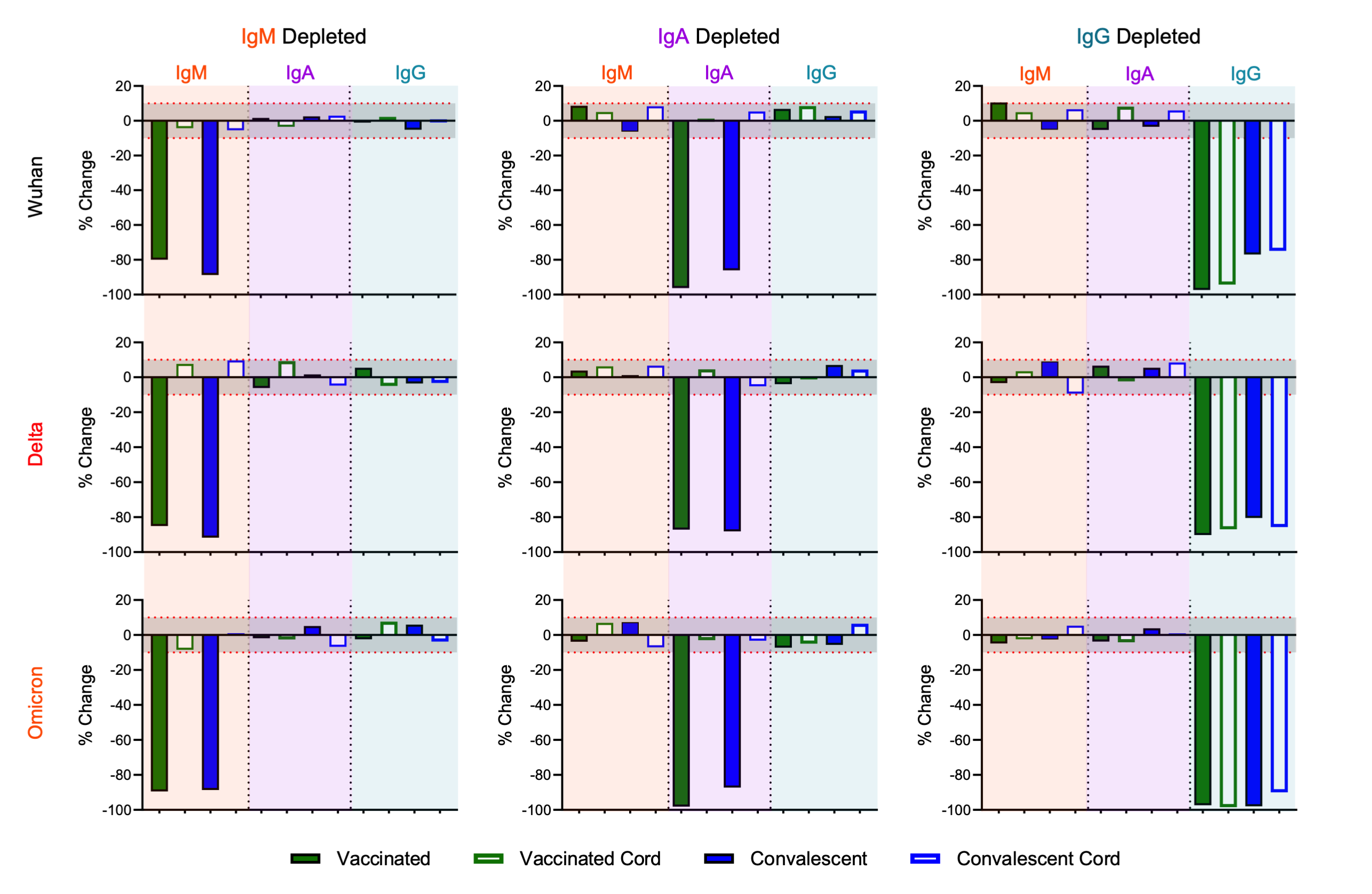

Supplement: S8 Fig — Maternal (filled) and cord (open) samples among convalescent (n = 15) (blue) or vaccinated (n = 15) (green) individuals were depleted of IgM (left), IgA (center), or IgG (right). Detection of antigen-specific immunoglobulin of each isotype was measured for each sample and percent change was calculated based on a mock control for each respective sample. Depletions were measured for antigen-specific antibodies to Wuhan (top), Delta (center), and Omicron (bottom) spike antigens. (TIFF) [file ppat.1013408.s008.tiff]

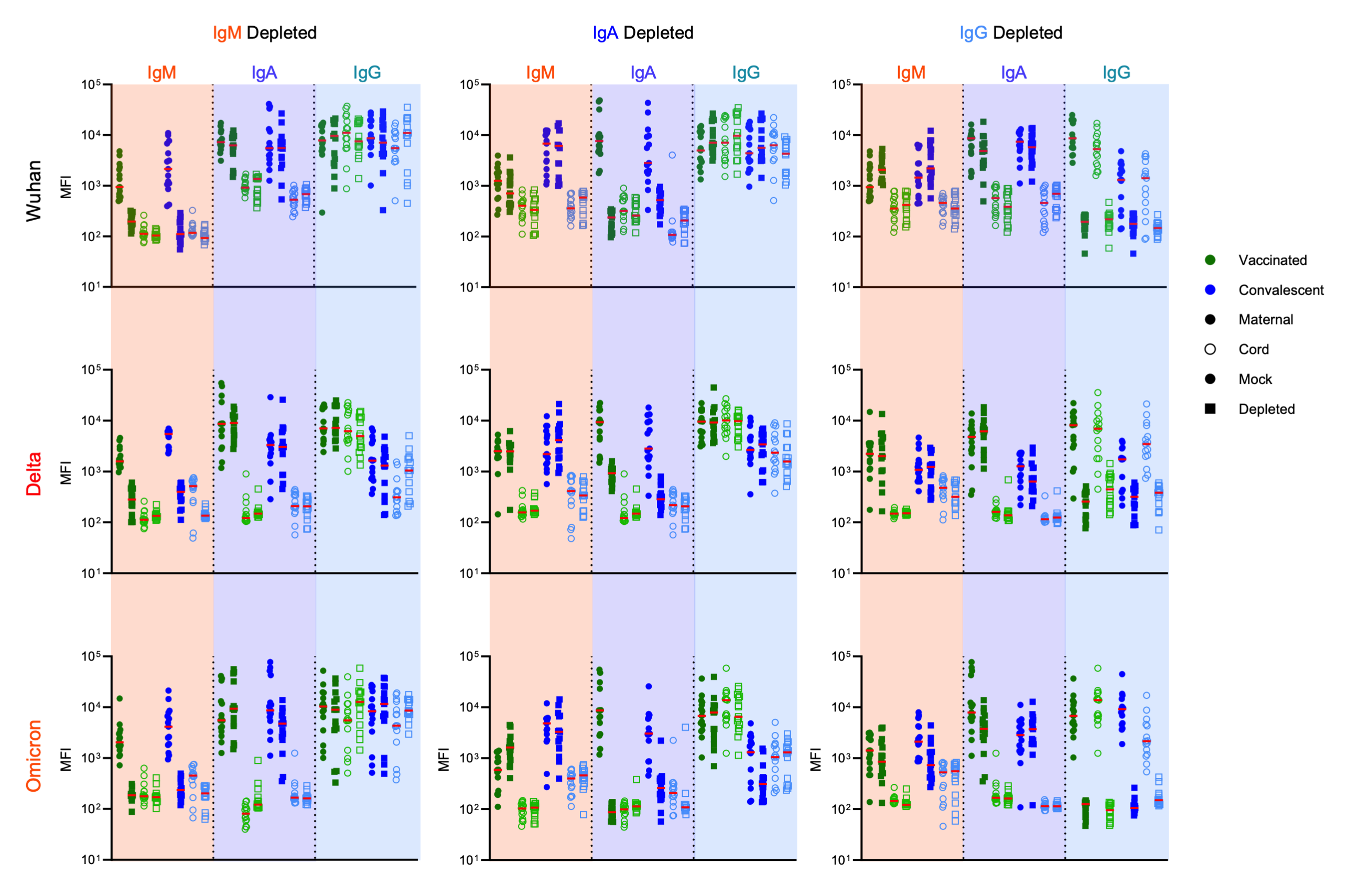

Supplement: S9 Fig — Maternal (filled) and cord (open) samples among convalescent (n = 15) (blue) or vaccinated (n = 15) (green) individuals were depleted of IgM, IgA, or IgG. Circle and square shapes denote mock and depleted samples, respectively. Binding was measured for each sample. Depletions were measured for antigen specific antibodies to Wuhan (top), Delta (center), and Omicron (bottom) spike antigens. Bar indicates median. (TIFF) [file ppat.1013408.s009.tiff]

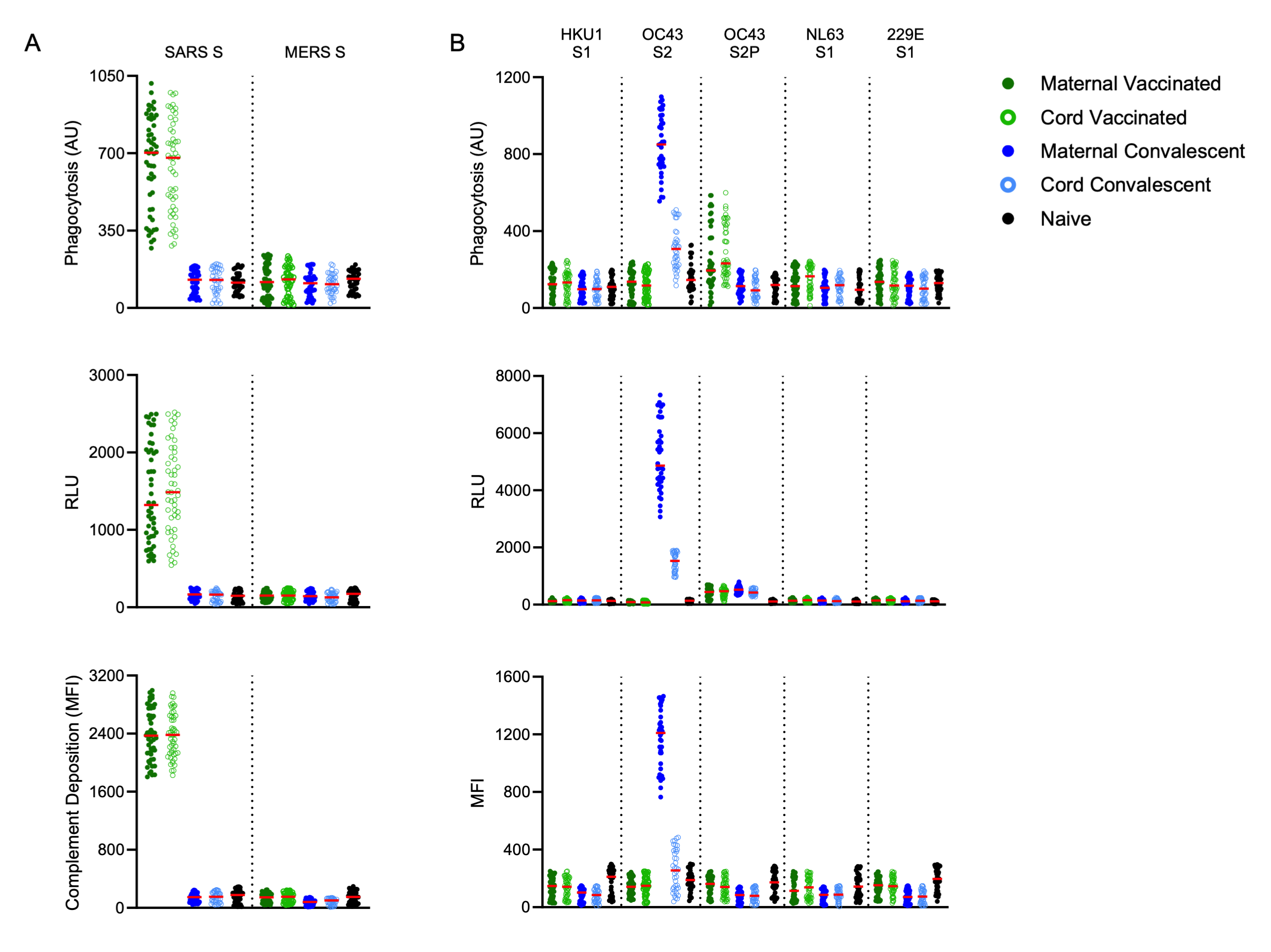

Supplement: S10 Fig — Fc effector functions against emergent coronaviruses SARS-CoV-1 and MERS-CoV (A) and endemic coronaviruses (B) HKU1, 0C43, NL63, and 229E. Maternal (filled) and cord (open) samples among convalescent (n = 38) (blue) or vaccinated (n = 50) (green) individuals were tested for ADCP (top), ADCC (center), and ADCD (bottom). Naïve subjects are shown in black (n = 37). Bar indicates median. (TIFF) [file ppat.1013408.s010.tiff]

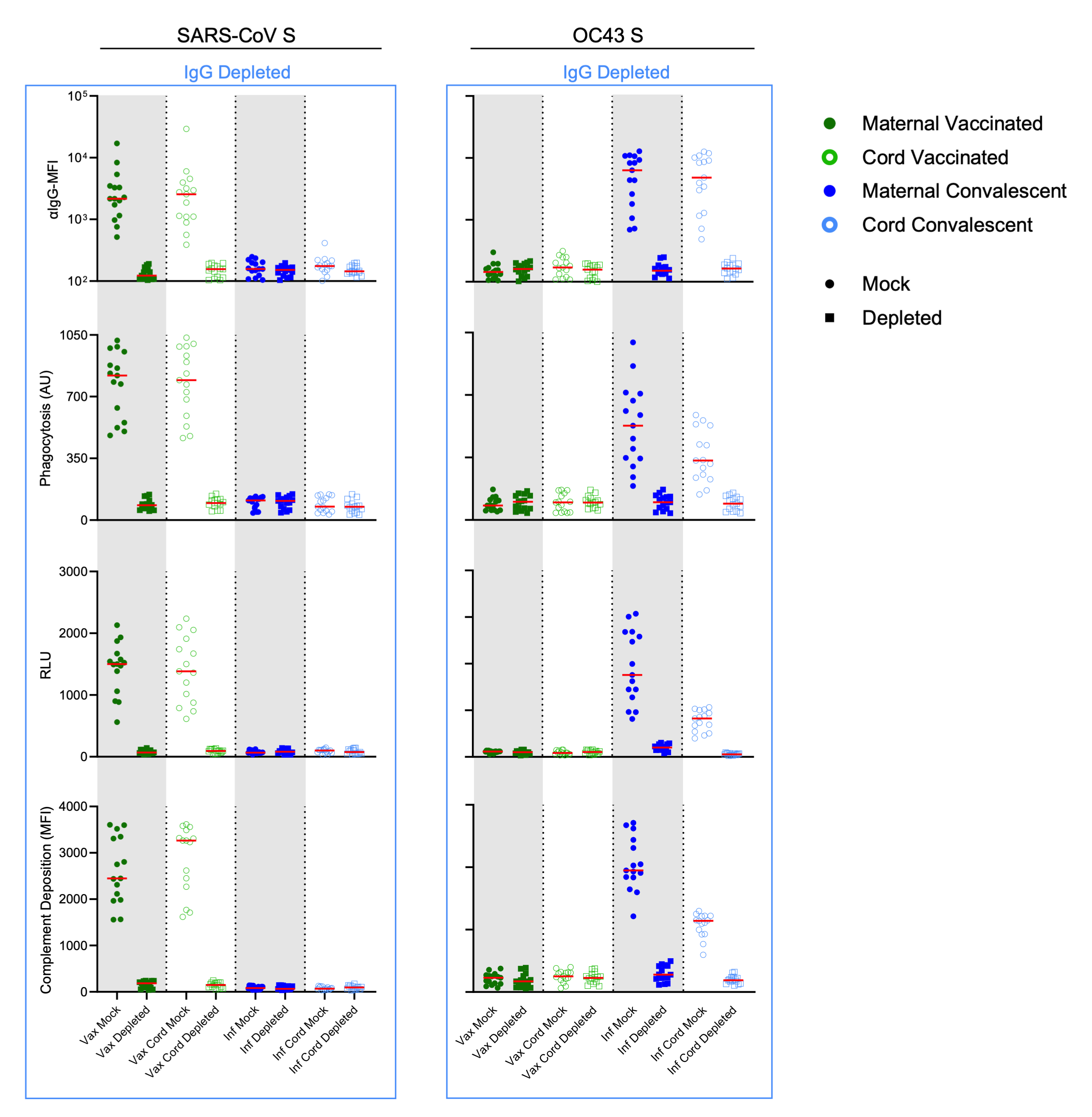

Supplement: S11 Fig — Maternal (filled) and cord (open) samples among convalescent (n = 15, blue) or vaccinated (n = 15, green) individuals were depleted of IgG and tested for IgG binding against SARS-CoV-1 S (left) and OC43 S (right). ADCP, ADCC, and ADCD effector functions were measured on depleted and mock control samples. Bar indicates median. (TIFF) [file ppat.1013408.s011.tiff]
